# Parental depressive symptoms and shared reading practices among preschool children in western China: cross-sectional study

**DOI:** 10.3389/fpsyt.2026.1957666

**Published:** 2026-09-21

**Authors:** Ziyi Wang, Hongli Sun, Jie Mi, Jiahua Liu

**Affiliations:** 1 School of Humanities and Social Science, Xi’an Jiaotong University, Xi’an, Shaanxi, China; 2 Shaanxi Institute for Pediatric Diseases, Xi’an Key Laboratory of Children’s Health and Diseases, Xi’an Children’s Hospital, Affiliated Children’s Hospital of Xi’an Jiaotong University, National Regional Children’s Medical Center (Northwest), Xi’an, Shaanxi, China; 3 Department of Pharmacy, Xi’an Children’s Hospital, Affiliated Children’s Hospital of Xi’an Jiaotong University, National Regional Children’s Medical Center (Northwest), Xi’an, Shaanxi, China; 4 Department of Clinical Laboratory, Xi’an Children’s Hospital, Affiliated Children’s Hospital of Xi’an Jiaotong University, National Regional Children’s Medical Center (Northwest), Xi’an, Shaanxi, China

**Keywords:** non-linear dose-response, parental depression, preschool children, shared reading, threshold effect

## Abstract

**Background:**

Shared reading supports early child development, but parental depressive symptoms may reduce its frequency and quality. Most previous studies assumed linear relationships with small samples. This large cross-sectional study examined linear and non-linear associations between parental depressive symptoms and shared reading practices in Chinese preschoolers.

**Methods:**

We recruited 21,366 parent-child dyads (children aged 3–6 years) from 189 kindergartens in western China. Depressive symptoms were measured with the Center for Epidemiologic Studies Depression Scale (CES-D). Shared reading was assessed using the StimQ READ subscale. Multivariable linear regression, piecewise linear regression with threshold estimation, and natural spline analysis were performed, adjusting for sociodemographic covariates.

**Results:**

Higher CES-D scores were linearly associated with lower total reading scores (adjusted β = –0.074 per point, 95% CI: –0.081 to –0.067). Piecewise regression identified a significant estimated breakpoint at CES-D score = 19.0 (95% CI: 11.9–26.1), below this threshold the slope was steeper (β = –0.085) than above (β = –0.051). Natural spline analysis confirmed non-linearity (P = 0.026) and located the point of maximum curvature at CES-D score = 10.9, indicating the most rapid change in slope occurred at subclinical symptom levels. The association was consistent across all subgroups (after Bonferroni correction).

**Conclusions:**

Parental depressive symptoms are negatively associated with shared reading in a non-linear, breakpoint-dependent manner. Even subclinical symptoms (CES-D score≈ 11) are associated with steeper declines, while the association attenuates at higher levels (CES-D score ≈ 19). Interventions targeting parental mental health, especially at subclinical to moderate symptom levels, may help preserve high-quality home literacy environments.

## 1 Introduction

Shared reading between parents and young children serves as a cornerstone of the home literacy environment (HLE) and plays a pivotal role in fostering early language, cognitive, and socio-emotional development. Joint book reading promotes vocabulary growth, print awareness, phonological sensitivity, narrative comprehension, and school readiness, with these positive effects often persisting into later academic achievement (1–3). Meta-analyses have consistently demonstrated that frequent and high-quality shared reading yields moderate to large benefits for children’s expressive and receptive language skills, particularly during the preschool years when neural plasticity for literacy-related abilities is pronounced (1, 4). These benefits extend beyond language domains to broader cognitive outcomes, including enhanced brain white matter integrity associated with cognitive abilities (3). Moreover, shared reading has been linked to improved emotional regulation (5), stronger parent-child bonding (6, 7), and reduced emotional and behavioral problems in young children (8, 9). Taken together, these multifaceted benefits position high-quality shared reading as one of the most valuable and modifiable components of a supportive early home environment.

Despite these well-established benefits, substantial variability exists in the frequency and quality of shared reading practices across families. Important barriers include socioeconomic constraints, limited access to books, time pressures, and parental psychosocial factors (10–12). Among these, parental depressive symptoms emerge as a particularly salient risk factor that can undermine responsive and stimulating parenting behaviors. Depression is associated with impaired emotional availability, motivation, and cognitive resources, and with reduced engagement in interactive activities such as shared reading, lower interaction quality, and diminished provision of literacy materials (13, 14). Evidence from randomized controlled trials further suggests that enhancements in parental psychosocial functioning and cognitive stimulation, including shared reading, can mediate positive effects on child behavioral outcomes (14). Thus, parental mental health represents a key modifiable influence on the quality of early home literacy practices.

Parental depressive symptoms represent a prevalent global public health concern. The Center for Epidemiologic Studies Depression Scale (CES-D) is a screening instrument that measures symptom severity rather than clinical diagnosis, and we therefore use “depressive symptoms” throughout to distinguish from clinically diagnosed depressive disorders. In China, rapid urbanization, socioeconomic pressures, and high parenting expectations contribute to notable rates of depressive symptoms among parents of young children (8, 15, 16). These challenges are particularly pronounced in western China, where economic development lags behind coastal regions and access to early childhood resources and mental health services remains limited (17). Both maternal and paternal depressive symptoms have been linked to suboptimal parenting practices, increased screen time, and adverse child developmental outcomes, while shared reading may serve as a potential protective factor (8, 18). These patterns highlight the importance of examining how parental depressive symptoms relate to early literacy environments, particularly in understudied populations such as western China.

A growing body of evidence indicates that parental depressive symptoms are associated with diminished HLE quality and reduced shared reading practices. Depressed parents tend to engage in less frequent and lower-quality shared book reading, which may partially mediate negative effects on children’s language and literacy development (8, 19, 20). Some studies further suggest potential bidirectional relationships, in which enriching HLE practices may help buffer parental stress and depressive symptoms, whereas reduced shared reading may exacerbate them (21, 22). However, existing studies have often been limited by relatively small sample sizes and have primarily focused on linear associations, with few investigations exploring potential non-linear dose-response relationships or thresholds in symptom severity. Unlike linear models that assume a constant decline, non-linear analysis can reveal thresholds at which the association between depressive symptoms and shared reading changes in magnitude. It remains unclear whether the association between depressive symptoms and shared reading is constant across the entire symptom spectrum, or whether specific ranges of depressive symptoms are associated with more pronounced changes in reading practices. Addressing this question could help determine whether intervention efforts should be broadly targeted or prioritized for parents within particular symptom ranges. Such thresholds could help identify symptom levels at which families might benefit from targeted support. Addressing these methodological limitations through large-scale analyses is therefore warranted.

The StimQ READ subscale offers an established, multidimensional parent-report tool that comprehensively assesses shared reading by evaluating reading quantity, concept diversity, content diversity, and interaction quality (3, 23, 24). The instrument has been used in published research with Chinese preschool populations across multiple subscales (25–27), supporting its applicability in this context. This instrument is particularly well-suited for examining nuanced associations between parental mental health and the home literacy environment. Its multidimensional structure facilitates a detailed evaluation of home literacy practices in large community-based samples.

The present large-scale cross-sectional study examines the associations between parental depressive symptoms (assessed with the CES-D) and shared reading practices (measured by the StimQ READ subscale) in a large community-based sample of 21,366 parent-child dyads recruited from public kindergartens in western China. We hypothesized that higher CES-D scores would be negatively associated with shared reading scores. In addition, we conducted exploratory analyses to examine whether this association might follow a non-linear pattern or exhibit a threshold effect. By clarifying these relationships, the current study aims to contribute additional evidence regarding the links between parental mental health and early literacy practices that may help inform culturally relevant public health strategies integrating parental mental health support with family literacy promotion.

## 2 Methods

### 2.1 Study design and participants

This cross-sectional investigation was conducted in collaboration with the local Education Bureau and participating schools in Yan’an, Shaanxi Province, western China. Stratified cluster sampling was employed, with the city’s 13 administrative districts serving as strata. From each district, public kindergartens were randomly selected using the official kindergarten roster, and the number drawn from each district was proportional to the district’s child population. All children aged 3–6 years and their parents were invited to participate between 28 February and 5 March 2025. One parent per child completed a self-administered questionnaire. The same parent completed all measures in the survey, including the CES-D and the StimQ READ subscale. The participating parent was the caregiver who was primarily responsible for the child’s daily care, and no restriction was placed on parent gender. Families decided which parent completed the questionnaire, typically based on availability and familiarity with the child’s routines. All eligible families in the selected kindergartens were invited, and no additional random sampling was applied within clusters. Of 28,000 distributed questionnaires, 25,017 were returned (89.3% response rate). After excluding responses with outlying parent age (n = 517), non-parental respondents (n = 2,408), and invalid child age (n = 726), the final analytical sample comprised 21,366 parent-child dyads.

### 2.2 Measures

#### 2.2.1 Parental depressive symptoms

Parental depressive symptoms were measured with the CES-D (28–30). This well-established self-report tool contains 20 questions that capture how often depressive symptoms occurred in the past week. Responses are given on a 4-point scale (0 = rarely or none of the time; 3 = most or all of the time). Summed scores span from 0 to 60, where higher values indicate more pronounced depressive symptomatology. Following the recommended screening cutoff of 16, parents were classified as having elevated depressive symptoms (score ≥16) or not (score <16) (31, 32). This cutoff has been validated in Chinese community populations and is widely used in studies of Chinese parents (32–34). The CES-D is a screening instrument, and this threshold serves to identify individuals at risk of depressive disorders rather than to establish a clinical diagnosis. In the current sample, the CES-D scale showed good reliability (Cronbach’s α = 0.85).

#### 2.2.2 Shared reading

Shared reading was assessed using the parent-reported StimQ, a well-established, ecologically valid tool originally developed to evaluate cognitive stimulation in children under 6 years old (35). For this study, we used a Chinese translation of the StimQ kindly provided by the original research team for research purposes. This translation has been used in published research with Chinese preschool children across multiple subscales (25–27). The StimQ comprises four subscales: reading activities (READ), parental involvement in developmental advancement (PIDA), parental verbal responsivity (PVR), and availability of learning materials (ALM) (35). For the purposes of this study, only the READ subscale was administered, which captures four core dimensions of shared reading: reading quantity, concept diversity, content diversity and interaction quality (3), which collectively assesses reading frequency, book availability, and interactive engagement during shared reading (36). This subscale consists mainly of yes/no items. The four reading dimensions are scored as follows: quantity (0–9), concept diversity (0–4), content diversity (0–4), and interaction quality (0–5), yielding a total reading score ranging from 0 to 22 (3, 23), with higher scores indicating a more supportive home literacy environment. In the present study, the total shared reading score and subscale scores were treated as continuous variables. In the current sample, the Cronbach’s alpha of StimQ was 0.882.

### 2.3 Covariates

The covariates were selected based on established literature on parental depression and parenting behaviors. Potential confounders included child age (years), child gender (boy/girl), parent age (years), parent gender (father/mother), education level (≤ junior high school, high school/junior college, ≥ undergraduate degree), occupation (working vs. not working), marital status (married/living with partner vs. others), and annual household income (< 30,000 ¥, 30,000–100,000 ¥, ≥ 100,000 ¥).

### 2.4 Statistical analysis

All analyses were performed with R version 4.5.2. Continuous variables are presented as mean ± SD, categorical variables as frequencies (percentages). Comparisons between parents with elevated depressive symptoms and parents without elevated depressive symptoms used t-tests (continuous) or chi-square tests (categorical).

Multivariable linear regression examined the associations of CES-D score (continuous) and depressive status (binary) with each reading outcome. The continuous analysis served as the primary approach to capture the full dose-response relationship, while the binary analysis (CES-D ≥16 vs. <16) was included as a secondary, complementary analysis to facilitate comparison with previous studies using this established screening cut-off. Two models were fitted: unadjusted (Model 1) and adjusted for all covariates (Model 2). Results are reported as unstandardized coefficients (β) with 95% confidence intervals (CI). Multicollinearity was assessed via variance inflation factors (VIF). All VIF values were < 2 (**Supplementary Table 1**).

To identify a potential threshold in the association between CES-D score and reading total score, we fitted a piecewise linear regression with one breakpoint using the segmented package. A single breakpoint was selected for several reasons. First, our primary aim was to test for the presence of a threshold effect rather than to identify multiple thresholds. A single breakpoint model provides a clear and interpretable starting point. Second, a parsimonious model with one breakpoint avoids overfitting and remains more directly interpretable for clinical and public health audiences. Models with multiple breakpoints were not systematically evaluated, as they would increase model complexity without a strong theoretical or empirical justification for multiple turning points in this context. The breakpoint (threshold) was estimated by maximum likelihood. Its 95% CI was derived by profile likelihood. A likelihood ratio test comparing the piecewise model with a simple linear model confirmed that the piecewise model fit significantly better (P for slope change = 0.0057). The significance of the slope change (difference between slopes below and above the threshold) was assessed using the z-test for the slope-difference term (U1.CES-D) provided by the segmented model.

A natural spline model with 4 degrees of freedom (knots at the 5th, 35th, 65th, and 95th percentiles of CES-D score) was fitted using the ns() function, adjusting for the same covariates. Non-linearity was tested by a likelihood ratio test against a linear model. The second derivative of the fitted spline was computed numerically, and the point of maximum curvature (i.e., the CES-D value where the second derivative of the fitted spline reaches its maximum absolute value) was identified. This metric describes the most rapid change in the slope of the dose-response curve and is presented as a statistical feature rather than a clinically meaningful threshold. Sensitivity analyses using 3 and 5 degrees of freedom were performed to assess robustness (**Supplementary Table 2**).

The primary analysis focused on the association between CES-D score and total reading score. Subscale analyses were secondary and interpreted descriptively using 95% CIs. We examined whether the association between depressive status and reading total score varied across subgroups defined by child age, child gender, parent age, parent gender, education, occupation, marital status, and household income. For each stratum, a linear regression model was run adjusting for all covariates except the stratification variable itself. Effect modification was tested by adding a product term (depressive status × stratum) to the full model. To account for multiple comparisons (8 tests), a Bonferroni correction was applied, setting the significance threshold at α = 0.00625 (0.05/8).

Kindergarten-level clustering was evaluated by the intraclass correlation coefficient (ICC) from a null mixed model and by cluster-robust standard errors. The ICC was 0.0015, and cluster-robust standard errors were nearly identical to ordinary standard errors (**Supplementary Table 3**), indicating negligible clustering effects. Given the negligible ICC, we retained ordinary regression as the primary model. Cluster-robust standard errors were prespecified as a sensitivity analysis to account for the clustered sampling design. To assess the potential impact of missing data, we performed multiple imputation on the full sample of 25,017 participants prior to applying any exclusion criteria. The imputation model included all variables used in the primary analysis. We used predictive mean matching with five imputations, which is suitable for continuous variables and does not require normality assumptions. Five imputations were considered sufficient given the stability of the pooled estimates. After imputation, the same exclusion criteria were applied. The pooled estimates were virtually identical to the complete-case results (**Supplementary Table 4**), indicating that missing data did not materially bias the findings. We assessed linear regression assumptions using standard diagnostic procedures: linearity via residual plots, homoscedasticity via the Breusch-Pagan test, influential observations via Cook’s distance, and residual distribution via Q-Q plots and the Shapiro-Wilk test. Results are summarized in **Supplementary Table 5**.

All tests were two-sided, and P < 0.05 was considered statistically significant unless otherwise specified.

## 3 Results

### 3.1 Participant characteristics

Among the 21,366 parents, 3,372 (15.8%) were classified as having elevated depressive symptoms (CES-D score ≥ 16). Compared with parents without elevated depressive symptoms, those with elevated depressive symptoms had higher CES-D scores (22.5 ± 6.8 vs. 9.1 ± 3.9, P < 0.001) and lower total reading scores (14.3 ± 3.7 vs. 15.6 ± 3.3, P < 0.001). Parents with elevated depressive symptoms were more likely to have lower education and lower household income, and to be unemployed (all P < 0.001). No significant differences were observed in child gender or parent gender (**Table 1**).

**Table 1:** Characteristics of the study population stratified by parental depressive status.

Characteristic	Overall (n=21366)	No elevated depressive symptoms (n=17994)	Elevated depressive symptoms (n=3372)	P-value
Parent age (years)	34.8 ± 4.6	34.7 ± 4.5	34.9 ± 4.7	0.008
Parent gender				0.437
Male	5108 (23.9)	4320 (24.0)	788 (23.4)	
Female	16258 (76.1)	13674 (76.0)	2584 (76.6)	
Child age (years)	4.8 ± 0.9	4.8 ± 0.9	4.9 ± 0.9	<0.001
Child gender, n (%)				0.483
Boys	11062 (51.8)	9305 (51.7)	1757 (52.3)	
Girls	10304 (48.2)	8689 (48.3)	1615 (47.7)	
Education level, n (%)				<0.001
≤ Junior high school	5741 (26.9)	4551 (25.3)	1190 (35.2)	
High school/Junior college	9476 (44.4)	8095 (45.0)	1381 (41.0)	
≥ Undergraduate	6149 (28.8)	5348 (29.7)	801 (23.8)	
Occupation, n (%)				<0.001
Not working	5724 (26.8)	4531 (25.2)	1193 (35.4)	
Working	15642 (73.2)	13463 (74.8)	2179 (64.6)	
Marital status, n (%)				<0.001
Married/Living together	20761 (97.2)	17546 (97.5)	3215 (95.3)	
Others	605 (2.8)	448 (2.5)	157 (4.7)	
Annual household income (¥), n (%)				<0.001
<30,000	8619 (40.3)	6925 (38.5)	1694 (50.2)	
30,000–100,000	8619 (40.3)	7349 (40.8)	1270 (37.7)	
≥100,000	4128 (19.3)	3720 (20.7)	408 (12.1)	
CES-D score	11.2 ± 6.6	9.1 ± 3.9	22.5 ± 6.8	<0.001
Read scale	15.4 ± 3.4	15.6 ± 3.3	14.3 ± 3.7	<0.001
Reading quantity	4.1 ± 2.1	4.2 ± 2.1	3.6 ± 1.9	<0.001
Concept diversity	3.7 ± 0.6	3.7 ± 0.6	3.6 ± 0.7	<0.001
Content diversity	3.2 ± 1.0	3.3 ± 1.0	3.1 ± 1.1	<0.001
Interaction quality	4.3 ± 1.2	4.4 ± 1.1	4.0 ± 1.4	<0.001

Data are mean ± SD or n (%). P-values from t-test (continuous) or chi-square test (categorical).

### 3.2 Linear associations

Higher CES-D scores were significantly associated with lower scores on all reading outcomes (all P < 0.001, **Table 2**). After full adjustment, each 1-point increase in CES-D score corresponded to a decrease of 0.074 points in the total reading score (95% CI: –0.081 to –0.067). To provide a more interpretable perspective on the magnitude of this association, a 5-point increase in CES-D score (e.g., from the sample mean of 11.2 to the screening cutoff of 16) corresponds to a 0.37-point decrease in reading score, which is approximately 0.11 SD of the reading score distribution. Similarly, parents with elevated depressive symptoms scored substantially lower than parents without elevated depressive symptoms on every reading measure. For example, the total reading score was 1.093 points lower in parents with elevated depressive symptoms (95% CI: –1.214 to –0.972, **Table 3**). Variance inflation factors were all below 2, indicating no multicollinearity (**Supplementary Table 1**). Cluster-robust standard errors for the CES-D coefficient (SE = 0.00393) were nearly identical to ordinary standard errors (SE = 0.00391), further supporting the use of ordinary regression as the primary model. The conclusions were unchanged in this sensitivity analysis (**Supplementary Table 3**).

**Table 2:** Linear regression of CES-D score on shared reading.

Outcome	Model 1 β (95% CI)	Model 2 β (95% CI)
**Read scale**	-0.091 (-0.098, -0.085)	-0.074 (-0.081, -0.067)
Reading quantity	-0.053 (-0.057, -0.048)	-0.038 (-0.043, -0.034)
Concept diversity	-0.002 (-0.004, -0.001)	-0.004 (-0.005, -0.003)
Content diversity	-0.012 (-0.014, -0.010)	-0.011 (-0.013, -0.009)
Interaction quality	-0.024 (-0.027, -0.022)	-0.021 (-0.024, -0.019)

Model 1: unadjusted. Model 2: adjusted for child age, child gender, parental education, occupation, marital status, household income, parental age, and parent gender. All P < 0.001.

Bold values denote the Read scale total score; non-bold values denote the four subscales.

**Table 3:** Linear regression of parental depressive status on shared reading.

Outcome	Depression status	Model 1 β (95% CI)	Model 2 β (95% CI)
Read scale	No elevated depressive symptoms	Ref.	Ref.
	Elevated depressive symptoms	-1.278 (-1.403, -1.154)	-1.093 (-1.214, -0.972)
Reading quantity	No elevated depressive symptoms	Ref.	Ref.
	Elevated depressive symptoms	-0.584 (-0.662, -0.507)	-0.450 (-0.525, -0.374)
Concept diversity	No elevated depressive symptoms	Ref.	Ref.
	Elevated depressive symptoms	-0.082 (-0.105, -0.059)	-0.096 (-0.118, -0.073)
Content diversity	No elevated depressive symptoms	Ref.	Ref.
	Elevated depressive symptoms	-0.216 (-0.253, -0.180)	-0.196 (-0.233, -0.160)
Interaction quality	No elevated depressive symptoms	Ref.	Ref.
	Elevated depressive symptoms	-0.395 (-0.438, -0.352)	-0.351 (-0.394, -0.308)

Model 1: unadjusted. Model 2: adjusted for child age, child gender, parental education, occupation, marital status, household income, parental age, and parent gender. All P < 0.001.

Bold values denote the Read scale total score; non-bold values denote the four subscales.

### 3.3 Threshold and non-linear dose-response

The piecewise linear regression revealed a significant statistical breakpoint at CES-D score = 19.0 (95% CI: 11.9–26.1). The P for slope change was 0.0057, which indicated that the slope below the threshold differed from the slope above it, supporting a change in the slope of the association at the estimated breakpoint (**Table 4**). Below this threshold, each 1-point increase in CES-D score was associated with a decrease of 0.085 points in the total reading score (95% CI: –0.096 to –0.075, P < 0.001). Above the threshold, the slope attenuated to –0.051 (95% CI: –0.073 to –0.029, P < 0.001), indicating that the decline in shared reading became shallower at higher symptom levels.

**Table 4:** Piecewise linear regression threshold for CES-D score on shared reading.

Outcome	Threshold	95% CI	Slope below threshold (95% CI)	P	Slope above threshold (95% CI)	P	P for slope change
Read scale	19.0	11.9 – 26.1	-0.085 (-0.096, -0.075)	<0.001	-0.051 (-0.073, -0.029)	<0.001	0.0057

Model adjusted for all covariates (as in **Table 2**). Threshold estimated by maximum likelihood; 95% CI from profile likelihood.

The natural spline model indicated a non-linear relationship (likelihood ratio test, χ²(3) = 9.26, P = 0.026). The estimated point of maximum curvature, a statistical feature of the fitted spline model, was 10.9 (**Figure 1**). This value should not be interpreted as a clinically meaningful threshold. Sensitivity analyses using 3 and 5 degrees of freedom yielded curvature points of 8.9 and 12.0, respectively (**Supplementary Table 2**).

**Figure 1:**
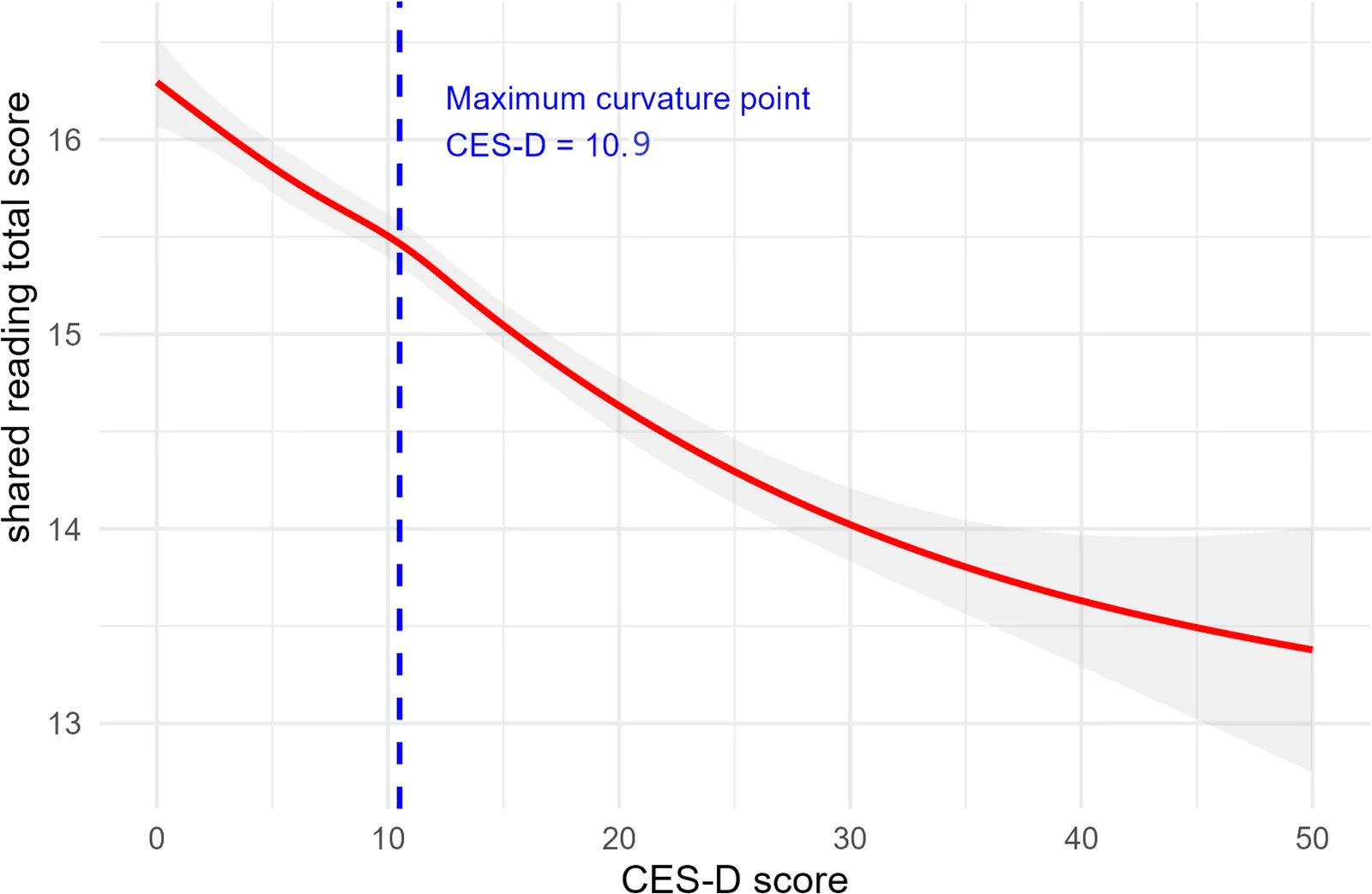
Natural spline curve of the association between CES-D score and shared reading total score. The model was adjusted for child age, child gender, parent age, parent gender, education level, occupation, marital status, and annual household income. The solid red line represents the predicted reading score, and the shaded gray area indicates the 95% confidence interval. The vertical blue dashed line marks the point of maximum curvature, located at CES-D score = 10.9, indicating the symptom level at which the slope of the decline changed most rapidly. This value is a statistical feature of the fitted curve and should not be interpreted as a clinically validated threshold. The natural spline model used 4 degrees of freedom (knots at the 5th, 35th, 65th, and 95th percentiles of CES-D).

### 3.4 Stratified analyses

The subgroup analyses did not show evidence of effect modification. None of the interaction terms reached statistical significance after Bonferroni correction for eight tests (raw P values ranged from 0.312 to 0.961, **Table 5**). Subgroup-specific estimates are presented in **Table 5** for descriptive purposes.

**Table 5:** Stratified analyses of the association between parental depressive status and shared reading.

Characteristics	Read scale		P interaction
	No elevated depressive symptoms	Elevated depressive symptoms	
		β (95% CI)	
Child age			0.422
<4	Ref.	-1.126 (-1.429, -0.823)	
≥4 to 5	Ref.	-0.987 (-1.205, -0.768)	
≥5	Ref.	-1.156 (-1.320, -0.992)	
Child gender			0.961
Boys	Ref.	-1.104 (-1.274, -0.933)	
Girls	Ref.	-1.081 (-1.253, -0.909)	
Parental age			0.835
<40	Ref.	-1.095 (-1.225, -0.965)	
≥40	Ref.	-1.081 (-1.415, -0.748)	
Parent gender			0.337
Male	Ref.	-1.170 (-1.418, -0.923)	
Female	Ref.	-1.073 (-1.212, -0.934)	
Education level			0.312
≤Junior high	Ref.	-0.981 (-1.204, -0.757)	
High school/Junior college	Ref.	-1.133 (-1.316, -0.950)	
≥Undergraduate	Ref.	-1.173 (-1.409, -0.936)	
Occupation			0.958
Not working	Ref.	-1.112 (-1.336, -0.889)	
Working	Ref.	-1.087 (-1.231, -0.942)	
Marital status			0.899
Married/Partner	Ref.	-1.092 (-1.216, -0.969)	
Others	Ref.	-1.263 (-1.923, -0.603)	
Household income (¥)			0.698
<30,000	Ref.	-1.143 (-1.322, -0.963)	
30,000–100,000	Ref.	-1.021 (-1.214, -0.828)	
≥100,000	Ref.	-1.138 (-1.458, -0.817)	

Models adjusted for all covariates except the stratification variable itself. All β were negative with P < 0.05. Interaction P-values are raw; after Bonferroni correction for 8 tests (α = 0.00625), none were significant.

## 4 Discussion

This large-scale cross-sectional study of 21,366 parent-child dyads recruited from public kindergartens in western China reveals a robust negative association between parental depressive symptoms and shared reading practices. Higher CES-D scores were significantly associated with lower total read scale scores as well as reduced scores on all four subscales, including reading quantity, concept diversity, content diversity, and interaction quality. A 5-point increase in CES-D score corresponds to a 0.37-point decrease in total reading score (approximately 0.11 SD), contextualizing the modest but statistically robust association. These relationships remained significant after comprehensive adjustment for child and parental sociodemographic characteristics. Parents with elevated depressive symptoms (CES-D score ≥16) demonstrated substantially lower engagement in shared reading activities compared with parents without elevated depressive symptoms. These results highlight the important association between parental mental health and the early home literacy environment (8, 18, 20).

A key advancement of the present study is the demonstration of non-linear dose-response patterns in the relationship between depressive symptoms and shared reading. Piecewise linear regression identified a statistically significant breakpoint at a CES-D score of 19.0, with a steeper negative slope below this point. The relatively wide confidence interval (11.9–26.1) indicates considerable uncertainty in the precise location of this breakpoint. This value should not be interpreted as a stable or clinically actionable cutoff. Rather, it is an estimated statistical feature of the dose-response curve. Replication in independent samples is needed to determine whether this breakpoint is reproducible across different populations. Sensitivity analyses using different natural spline degrees of freedom yielded consistent curvature points (8.9–12.0), supporting the robustness of the overall non-linear pattern. Taken together, these findings suggest that the overall trend, a steeper decline in shared reading at lower depressive symptom levels, is more robust than the exact breakpoint value itself. Complementary natural spline analysis confirmed non-linearity, with the point of maximum curvature occurring at approximately 10.9. This value is a statistical feature of the fitted dose-response curve and should not be interpreted as a clinically meaningful cutoff. These findings indicate that even relatively subclinical levels of depressive symptoms may be associated with disruption of shared reading practices. The observed attenuation at higher CES-D levels may reflect a true plateau in the association, but could also be partly influenced by the distribution of high scores, measurement characteristics, or model specification. Accordingly, this pattern should be interpreted with appropriate caution. Unlike most previous studies (8, 14, 19) that primarily relied on linear models and smaller samples, the current study adds to the literature by systematically examining threshold effects and non-linear patterns in the association between parental depressive symptoms and home literacy practices. This nuanced characterization provides deeper insight into the dose-response relationship and raises the possibility that intervention efforts may be particularly impactful when targeting subclinical to moderate depressive symptoms.

The estimated breakpoint from piecewise regression (19.0) and the point of maximum curvature from natural spline (10.9) represent distinct statistical features. The breakpoint indicates a structural change in the slope of the association, while the point of maximum curvature marks where the slope of the dose-response curve changed most rapidly. These two values are not competing thresholds but complementary aspects of the same non-linear pattern: the decline is steepest in the subclinical-to-moderate range and attenuates after approximately 19.0. Neither value should be interpreted as a clinically validated threshold; both are statistical features of their respective models and should be interpreted with appropriate caution.

Several mechanisms may plausibly explain the observed associations, though they were not directly tested in this study and remain speculative. Depressive symptoms may be associated with reduced parental emotional availability, cognitive flexibility, and motivation for sustained interactive engagement (20, 37, 38), which in turn may contribute to lower frequency and quality of shared book reading. Parents experiencing depression may be less likely to use dialogic reading strategies, such as asking open-ended questions or expanding upon story narratives, which are particularly beneficial for child development (39, 40). Furthermore, depressive symptoms may be accompanied by household disorganization and heightened parenting stress (41, 42), which could further limit opportunities for consistent literacy activities. Bidirectional pathways are also plausible. Lower levels of enriching shared reading may increase parental feelings of inefficacy and stress, potentially worsening depressive symptoms over time (21, 43, 44). However, these hypothesized pathways were not empirically tested in this study and should be interpreted as hypotheses for future research.

The negative association between parental depressive status and shared reading was remarkably consistent across multiple sociodemographic subgroups, including variations in child age, gender, parental education, occupation, marital status, and household income. No significant effect modification was observed after applying a stringent Bonferroni correction. This broad pattern suggests that parental depression may function as a pervasive rather than context-specific risk factor for diminished home literacy practices. Nevertheless, the absence of significant effect modification may reflect several possibilities. First, statistical power for detecting interaction effects may have been limited in some strata, particularly those with smaller subgroup sizes. Second, the association between depressive symptoms and shared reading may indeed be consistent across these sociodemographic characteristics. Third, residual confounding from unmeasured psychosocial factors (e.g., parenting stress, social support) may have contributed to the null interaction findings. These possibilities should be considered when interpreting the subgroup results. In the context of China’s rapid socioeconomic transitions and intensive parenting norms, these findings highlight the need to address parental mental health within broader efforts to promote equitable early childhood development (8, 15).

From a developmental perspective, the reduction in shared reading associated with parental depressive symptoms may be linked to cascading consequences for children. Prior literature has shown that high-quality shared reading supports not only language and cognitive skills but also socio-emotional competencies through responsive parent-child interactions and opportunities for emotional co-regulation (9, 45). When such activities are limited, children may have fewer opportunities for vocabulary enrichment, narrative comprehension, and secure attachment formation, which could be associated with developmental disparities during a highly sensitive period (46–48). These pathways are consistent with previous research but were not directly assessed in the current study. Nevertheless, these results suggest that parental mental health and home literacy practices may be interconnected targets for intervention, though this hypothesis requires evaluation in future intervention studies.

### 4.1 Strengths and limitations

This study has several notable strengths. It features one of the largest community-based samples to date on this topic, a high response rate of 89.3%, the use of well-validated instruments for both depressive symptoms and shared reading, rigorous covariate adjustment, and advanced statistical approaches capable of detecting non-linear patterns. Multiple sensitivity analyses further support the robustness of the findings. Nevertheless, several limitations should be acknowledged. First, the cross-sectional design precludes causal inferences about directionality, bidirectional or third-variable influences cannot be ruled out. Second, a key limitation is that all measures relied on parent self-report, and both the exposure (depressive symptoms) and the outcome (shared reading) were reported by the same parent. This introduces the potential for same-rater bias and common-method bias, which may inflate the observed associations. Depressed parents may underreport positive parenting behaviors due to negative cognitive bias or reduced self-efficacy, which could lead to an overestimation of the negative association between depressive symptoms and shared reading. This should be considered when interpreting the magnitude of the observed associations. Moreover, the CES-D is a screening tool rather than a clinical diagnostic instrument, and the StimQ has not been formally validated in mainland Chinese populations. Additionally, the shared reading total score ranges from 0 to 22, which may limit variability at the extremes and potentially attenuate observed associations. Third, the sample, while large, was drawn from public kindergartens in one western Chinese city, limiting generalizability to rural populations or other cultural contexts. Future studies in diverse Chinese populations are needed to establish the external validity of these findings. Additionally, although the response rate was high (89.3%), participating parents may differ systematically from non-participants, for example in their level of engagement in child-rearing or literacy activities. This could affect the generalizability of the findings to families with lower engagement or different caregiving arrangements, and should be considered when interpreting the results. Fourth, despite adjusting for multiple sociodemographic covariates, residual confounding by unmeasured factors (such as parenting stress, social support, household chaos, access to books, child temperament, and screen exposure) remains possible. Fifth, the estimated breakpoint (CES-D = 19.0) should be interpreted with caution. Its relatively wide confidence interval (11.9–26.1) indicates considerable uncertainty, and this value was derived from a single sample. Replication in independent samples is needed before any clinical or public health interpretation can be considered.

## 5 Conclusion

This large cross-sectional study of 21,366 parent-child dyads in western China demonstrates a significant negative association between parental depressive symptoms and shared reading practices. The association was non-linear, with the decline being more pronounced at lower symptom levels and attenuating at higher levels. These findings suggest that even subclinical depressive symptoms may be relevant to home literacy environments. Given the cross-sectional design, the results should be interpreted as associations rather than causal effects. Future longitudinal and intervention studies are needed to clarify directionality and to evaluate whether parental mental health may represent a potential target for integrated early-childhood interventions aimed at supporting home literacy practices.

## Data Availability

This data set is still being used for analysis. Please contact the corresponding author regarding access to the full dataset. Requests to access the datasets should be directed to Jiahua Liu, liujiahua698@163.com.
