# Supplementary material for: Parental depressive symptoms and shared reading practices among preschool children in western China: cross-sectional study
Source: Front Psychiatry. 2026 Sep 21;17:1957666. doi: 10.3389/fpsyt.2026.1957666 (PMC13635101; doi:10.3389/fpsyt.2026.1957666)
Supplement: Supplementary file 1 [file Table1.docx]

Supplementary Table S1. Variance inflation factors（VIF）

| **Variable** | **VIF** |
| --- | --- |
| CES-D | 1.024 |
| Child age | 1.015 |
| Child gender | 1.001 |
| Education level | 1.071 |
| Occupation | 1.150 |
| Marital status | 1.012 |
| Household income each year | 1.033 |
| Age | 1.031 |
| Parent gender | 1.058 |

All VIF < 2, indicating no multicollinearity.

Supplementary Table S2. Sensitivity of natural spline curvature point to degrees of freedom(df)

| **Degrees of freedom** | **AIC** | **BIC** | **Maximum curvature point (CES-D)** |
| --- | --- | --- | --- |
| 3 | 111035.5 | 111155.1 | 8.9 |
| 4 | 111035.6 | 111163.2 | 10.9 |
| 5 | 111024.4 | 111159.9 | 12.0 |

The point of maximum curvature (where the slope of the decline changes most rapidly) varies from 8.9 to 12.0 across df = 3–5, all falling within the mild depressive symptom range (CES‑D < 16), indicating robustness. Df = 4 was selected as the primary analysis because it represents a trade‑off between model fit and parsimony (AIC favours df = 5, BIC favours df = 3) and is the most frequently used setting in the literature.

Supplementary Table S3. Kindergarten clustering assessment

| **Between‑kindergarten variance** | **Within‑kindergarten variance** | **ICC** | **Number of kindergartens** |
| --- | --- | --- | --- |
| 0.0031 | 2.0136 | 0.0015 | 189 |

ICC = between‑kindergarten variance / (between‑kindergarten variance + within‑kindergarten variance). The ICC was essentially zero, indicating no meaningful clustering by kindergarten.

Supplementary Table S4. Sensitivity analysis of missing data: comparison of complete‑case and multiple imputation linear regression estimates

| **Analysis** | **N** | **β (95% CI)** |
| --- | --- | --- |
| Complete‑case | 21,366 | -0.074 (-0.081, -0.067) |
| Multiple imputation (5 imputations) | 25,017 | -0.074 (-0.081, -0.067) |

Multiple imputation was performed using predictive mean matching (PMM) with 5 imputations. Estimates were pooled using Rubin’s rules. The two sets of estimates are virtually identical, indicating that missing data did not bias the findings.

Supplementary Table S5. Diagnostic checks for linear regression assumptions

| **Assumption** | **Method** | **Result** |
| --- | --- | --- |
| Linearity | Residual plots | No apparent nonlinear patterns |
| Homoscedasticity | Breusch-Pagan test | χ² = 209.75, P < 0.001* |
| Normality of residuals | Shapiro-Wilk test (n=5000) | W = 0.9787, P < 0.001* |
| Influential observations | Cook‘s distance | Maximum = 0.0041 |
| Model fit | R² / Adjusted R² | 0.0967 / 0.0962 |

Note: The Breusch-Pagan and Shapiro-Wilk tests were significant, which is expected given the large sample size (n = 21,366). Visual inspection of residual and Q-Q plots did not reveal substantial deviations. Linear regression is robust to mild deviations from these assumptions with such a large sample. The maximum Cook’s distance was well below the conventional threshold of 1, indicating no influential observations.
